# Abstract of Proceedings of the Buffalo Medical Association

**Published:** 1864-12

**Authors:** Joseph A. Peters

**Affiliations:** Secretary


ART. III.—*Abstract of Proceedings of the Buffalo Medical Association.*

TUESDAY EVENING, November 1, 1864.

Society met pursuant to adjournment, the President, Dr. Samo, being in the Chair.

Present—Drs. White, Wyckoff, Cronyn, Strong, Shaw, Congar, Wetmore, Ring, Miner, Smith, Gay and Peters.

Dr. J. C. Greene was proposed as a member.

There being no "voluntary communications" presented, "reports on prevailing diseases" were called for.

*Dr. Shaw* reported having noticed a prevailing typhoid character in all forms of disease.

*Dr. Wetmore* had remarked the same tendency. Also gave the outline of several cases of obstinate intermittent fever among the soldiers at Fort Porter, which had resisted all forms of treatment thus far adopted. Had tried quinine, Fowler's Solution, (until constitutional effects of arsenic were produced,) acetate of potash, etc., but nearly two-thirds of the cases still had intermittent, some having true ague, and some a marked form or "dumb ague."

*Dr. White* thought there was no epidemic prevailing of any sort. The late autumnal diseases, such as pulmonary and laryngeal difficulties, seemed to be replacing bowel complaints, while the low type of disease, as usual at this season, was disappearing. Thought the city on the whole very healthy. In reference to Dr. Wetmore's cases would say he had tried, in similar cases, valerianate of zinc, as well as biberine and sulphate of zinc, (the latter without much effect,) with sometimes good results. The hypodermic injection of quinine often answered a good purpose when the stomach would not bear the remedy.

A desultory discussion here took place between Drs. White, Miner and Wyckoff, in regard to the relative safety of the sulphate, and the acetate of morphia as a preparation for hypodermic injection, wherein the weight of experience seemed to be in favor of the harmlessness of the sulphate.

After the transaction of some miscellaneous business the Association adjourned. JOSEPH A. PETERS, Secretary.

